# Glycemic control and its associated factors among children and adolescents with type 1 diabetes in Ethiopia: a narrative review

**DOI:** 10.11604/pamj.2026.54.5.50512

**Published:** 2026-05-07

**Authors:** Yemane Leake, Hindeya Hailu Hagos, Birhanu Kassie Reta

**Affiliations:** 1 Department of Pediatrics and Child Health, College of Health Sciences, Aksum University, Aksum, Ethiopia,; 2 College of Health Sciences, School of Medicine, Aksum University, Aksum, Ethiopia

**Keywords:** Diabetes mellitus, children, adolescents, glycemic control, narrative review, Ethiopia

## Abstract

Glycemic control is a critical component in the management of diabetes mellitus among children and adolescents. This narrative review synthesizes existing evidence on the status of glycemic control-both good and poor-and its associated factors among Ethiopian children and adolescents living with diabetes. The objective of the review was to assess the pattern of glycemic control and identify factors associated with both good and poor glycemic outcomes among children and adolescents with diabetes in Ethiopia. Relevant studies reporting on glycemic control and associated factors were identified through searches conducted in Google Scholar, PubMed/MEDLINE, African Journals Online, and the Ethiopian Medical Journal. Additional grey literature sources were also reviewed. Eligible studies were screened and narratively synthesized. The prevalence of poor glycemic control among Ethiopian children and adolescents ranged from 39.3% to 89.3%. Conversely, good glycemic control was reported in 16.4% to 60.7% of participants. Factors associated with glycemic control included socio-demographic characteristics, treatment-related variables, health system constraints, and dietary habits. Poor glycemic control is widespread among Ethiopian children and adolescents with diabetes, underscoring the urgent need for improved management strategies and targeted interventions. Strengthening diabetes education, promoting self-monitoring of blood glucose, and improving access to essential medical supplies and insulin may help enhance glycemic outcomes.

## Introduction

Diabetes mellitus is a complex metabolic disorder characterized by chronic hyperglycemia resulting from defects in insulin secretion, insulin action, or both [1-3]. Inadequate insulin production and/or impaired tissue responsiveness to insulin lead to insufficient insulin activity at target sites, causing disturbances in carbohydrate, fat, and protein metabolism [1,3]. In children and adolescents, the diagnosis of diabetes is based on one or more of the following criteria: the presence of classic symptoms or a hyperglycemic crisis with a plasma glucose level ≥ 11.1 mmol/L (200 mg/dL); a fasting plasma glucose ≥ 7.0 mmol/L (126 mg/dL), defined as no caloric intake for at least 8 hours; a two-hour plasma glucose = 11.1 mmol/L (200 mg/dL) during an oral glucose tolerance test (OGTT); or a glycated hemoglobin (HbA1c) level ≥ 6.5% [1,3]. Diabetes mellitus in children encompasses several types, including type 1 diabetes, type 2 diabetes, monogenic diabetes, and other uncommon forms such as cystic fibrosis-related and drug-induced diabetes [2,3]. Type 1 diabetes is the most prevalent form in the pediatric population comprising > 90%, while monogenic diabetes accounts for a small but distinct subset of cases. The incidence of type 1 diabetes varies considerably across geographic regions and is influenced by factors such as age, ethnicity, gender, and family history [4]. In Ethiopia, the burden of diabetes among children and adolescents is increasing at an estimated annual rate of 3-4% [4]. Despite this growing trend, diabetes care for the pediatric population remains limited, highlighting the urgent need to achieve glycemic targets and strengthen diabetes management for children and adolescents in the country.

According to the International Diabetes Federation's 2021 estimates, approximately 108,300 children and adolescents under the age of 15 and 149,500 under the age of 20 were living with type 1 diabetes worldwide [5]. Furthermore, in 2021, there were an estimated 651,700 children and adolescents living with the condition worldwide. Management of diabetes in children and adolescents requires specialized and developmentally appropriate care due to unique challenges such as fluctuating insulin sensitivity associated with physical growth and puberty, varying levels of self-care capacity, the need for supervision in home and school settings, increased neurological vulnerability to both hypo- and hyperglycemia, and their potential neurocognitive consequences [6].

Optimal glycemic control plays a crucial role in preventing both acute and long-term complications among children and adolescents with diabetes [6]. Poor glycemic control is strongly associated with acute complications-including diabetic ketoacidosis and severe hypoglycemia-as well as chronic vascular complications such as retinopathy, nephropathy, and neuropathy [1,7,8]. Beyond medical consequences, poor glycemic control also carries significant social, behavioral, economic, and psychological impacts over time. Achieving and maintaining optimal glycemic levels is therefore considered one of the most important factors in reducing the risk of complications in young people living with diabetes [9]. A recently published systematic review in Ethiopia reported a pooled prevalence of poor glycemic control of 71% [10] among children with type 1 diabetes, underscoring the magnitude of the problem at the national level. Although a few single-center studies and aggregated datasets have examined glycemic control in this population, comprehensive national evidence remains scarce. This narrative review brings together the existing evidence on glycemic control patterns and their associated factors among children and adolescents living with diabetes in Ethiopia. By summarizing current findings, the review aims to support policymakers, diabetes associations, and healthcare providers with evidence-based insights that can help strengthen diabetes management, address ongoing challenges, and ultimately improve outcomes for this vulnerable population.

## Methods

### Search strategy

A comprehensive literature search was conducted to identify studies reporting on glycemic control and associated factors among children and adolescents with diabetes in Ethiopia. Due to variability in the definitions of poor versus good glycemic control across different guidelines and professional associations over time, this study relied on the definitions used during the respective study periods. Most studies defined glycemic control using an HbA1C cutoff of 7.5%, while one study used a cutoff value of 7%. The databases searched included PubMed, Google Scholar, African Journals Online, and the Ethiopian Medical Journal. The final search was performed on October 20, 2025. Grey literature sources were also reviewed to capture relevant unpublished or non-indexed studies. The search was limited to articles published in English, with no restriction on publication year. Key search terms included: (“diabetes mellitus” OR “type 1 diabetes” OR “pediatric diabetes”) AND (children OR adolescents OR pediatrics) AND (“glycemic control” OR “poor glycemic control” OR “good glycemic control” OR “blood glucose level” OR “serum glucose level” OR HbA1c) AND (Ethiopia OR “resource-limited setting” OR “low-income country”). Boolean operators (AND, OR, NOT) were applied to refine the search.

### Inclusion and exclusion criteria

**Figure 1:**
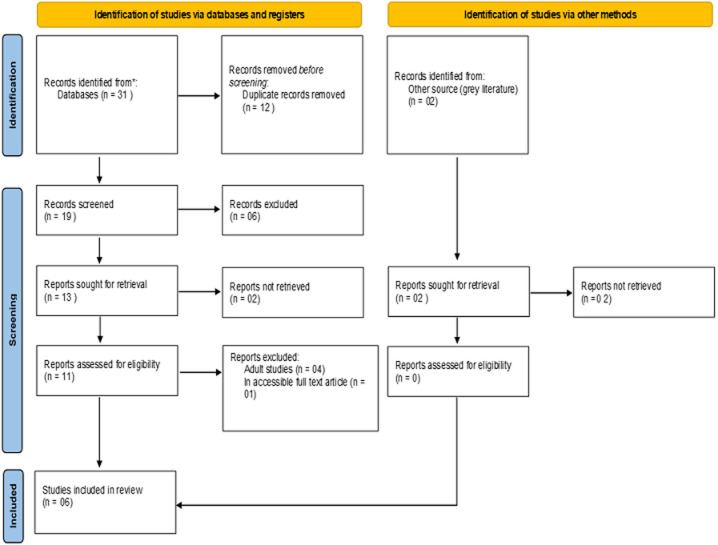
study selection flow diagram

Studies were included if they met the following criteria: Conducted in Ethiopia, Focused on children and adolescents with diabetes mellitus (aged < 20 years), Reported on glycemic control and/or associated factors, Published in English and Available in full text; otherwise excluded. All types of study designs reporting glycemic control among children and adolescents were eligible for inclusion. A summary of the selection process is presented in Figure 1.

### Data extraction and synthesis

Articles were initially screened based on titles and abstracts. When the information provided was insufficient to determine eligibility, the full-text version was retrieved and assessed. Screening was guided by the following criteria: title relevance, abstract content, language (English), participant age (children and adolescents <20 years), and study location (Ethiopia). For each included study, the following data were extracted and summarized: participant age, glycemic control status (categorized as good or poor), study area, study design, sample size, HbA1C levels, and factors associated with glycemic control. YL and HH screened and extracted the data, and BK was consulted when disagreements occurred. Table 1 provides a summary of the studies included in this review.

**Table 1:** summary of studies included in the review

Author, year of publication	Study population	Type of diabetes	Sample size	Study area	Study design	Mean HbA1c	HbA1c Cutoff values	Result in %		Factors associated with glycemic control
								Poor	Good	
Meseret *et al*. [10]	Pediatric	Type 1	1380	-	Systematic review and meta-analysis			71%	NA	Having glucometer
Habteyohans *et al.*, 2023 [11]	1-18year	Type 1	231	Eastern Ethiopia	Cross sectional	10.4	7.5%	71.9	28.1	Age, Educational level of caregiver, Consumption of forbidden foods, and meal frequency
Shibeshi *et al*., 2022 [12]	21 mon-18 years	Type 1	116	Southern Ethiopia	Cross sectional	9.6 ± 2.4	7.5%	83.6	16.4	Lipodystrophic changes at injection sites, could not afford to buy insulin
Kidie *et al*., 2025 [13]	< 18 years	Type 1	389	Northwest		NA, instead, FBS and RBS were used	7%	39.3	60.7	Age, treatment discontinuation, and doses of treatment
Adege *et al*., 2025 [14]	<18	Type 1	203	North east Ethiopia	Cross sectional	NA	7.5%	47.3	52.7	Being third born, diabetic complications and other comorbid conditions
Abrahim *et al*., 2023 [15]	≤ 18 years	Type 1	158	Southwest Ethiopia	Cross sectional	9.67 ± 2.28%	7.5%	76.6	23.4	Caregiver relationship, insulin injection supervision, problems during hospital care, Blood glucose monitoring, previous admission

## Results

The search process identified 33 studies from electronic databases. After removing 14 duplicates and excluding 8 articles deemed irrelevant based on their titles and abstracts, 11 full-text articles were assessed for eligibility. Of these, 4 articles were excluded for not meeting the inclusion criteria, and 1 was excluded due to inaccessibility. Ultimately, 6 studies were included in the final review (Figure 1). The final review comprised five cross-sectional studies and one systematic review with meta-analysis [10-15]. Four of the five cross-sectional studies were single-centered and conducted in referral and teaching hospitals, while one study was conducted across two referral hospitals. All study settings were in urban areas. In the five reviewed articles, the sample sizes ranged from 116 to 389 participants in individual studies, totaling 1,097 participants across the cross-sectional studies. The sixth article (the systematic review and meta-analysis) included 1,380 participants [10].

All included studies consistently reported a high prevalence of poor glycemic control, primarily measured by HbA1c, among Ethiopian children and adolescents with diabetes mellitus. The proportion of participants with poor glycemic control ranged from 39.3% to 89.3%. Conversely, the prevalence of good glycemic control ranged from 16.4% to 60.1%. All studies were conducted in referral and teaching hospitals located in various regions of Ethiopia, including eastern, northeastern, southern, western, and northwestern areas. No studies reporting glycemic control from the Tigray region-the northernmost part of the country-were identified.

Each study also examined factors associated with glycemic control. The most commonly reported factors linked to poor glycemic control included: Socio-demographic factors: age of the patient, birth order, educational status of the caregiver and child, family income, residence, and sex of the primary caregiver, Behavioral and adherence-related factors: consumption of forbidden foods, which refers to food items not recommended for diabetic patients except in times of hypoglycemia, meal frequency, history of hospital admission, and history of treatment discontinuation, treatment-related factors: presence of diabetic complications and comorbidities, lipodystrophic changes at insulin injection sites, inability to afford insulin when not freely available, insulin dosage, and caregiver-patient relationship, health system factors: challenges during hospital care, irregular blood glucose monitoring, and limited availability of glucometers and Psychosocial factors: lack of family involvement in insulin administration and supervision [10-15].

In contrast, certain factors were associated with good glycemic control, including higher insulin dosage (per unit body weight) and preschool age. Interestingly, one study reported that children with diabetic complications had better glycemic control, a finding contrary to expectations [14].

## Discussion

This narrative review provides a comprehensive assessment of glycemic control and its associated factors among children and adolescents with diabetes in Ethiopia. Most of the included studies were facility-based cross-sectional studies, along with one systematic review and meta-analysis, conducted across multiple regions and involving individuals under 20 years of age (Table 1). The primary outcome assessed was glycemic control, categorized as either good or poor. The review revealed that the prevalence of poor glycemic control among Ethiopian children and adolescents ranged from 39.3% to 89.3% [10-15]. All studies consistently reported a high rate of inadequate glycemic control, while only a minority of children achieved relatively good control. This underscores the limitations of the Ethiopian healthcare system in providing optimal diabetes care for children and adolescents. Comparable findings have been reported in other African countries: a Tanzanian study found that most participants had poor glycemic control [16], while a study in Sudan indicated that over two-thirds of participants had poor control, with fewer than a quarter achieving good control [17]. In Kenya, Ngwiri *et al*. reported that 72% of children and adolescents had poor glycemic control, using an HbA1c cutoff of 8% [18].

Achieving glycemic targets in children and adolescents is critical, particularly in resource-limited settings such as Ethiopia. Access to pediatric diabetes care remains highly centralized and is primarily available at referral and teaching hospitals [6]. In addition, there is a shortage of essential glucose monitoring equipment, including glucometers [10] and HbA1c analyzers, as well as limited insulin availability. These challenges are further compounded by a scarcity of healthcare professionals trained in pediatric diabetes care. Consequently, the findings of this review support these observations. Type 1 diabetes mellitus is the most common form of diabetes in children [1,4]. All primary studies and the systematic review included in this review were conducted among children with type 1 diabetes. A search was also performed for studies conducted on type 2 diabetes in children in Ethiopia; however, no eligible studies were identified. Pediatric diabetes management requires specialized care due to physiological changes such as fluctuating insulin sensitivity during growth and puberty, variable ability to perform self-care, the need for supervision at home and school, neurological vulnerability to hypo- and hyperglycemia, and potential neurocognitive effects [6].

According to the American Diabetes Association (ADA) 2024 guidelines, HbA1c targets should be individualized. Generally, an HbA1c of < 7% is recommended, with a less stringent target of < 7.5% for children who cannot reliably recognize hypoglycemia. Selected individuals may aim for < 6.5% if tight control is safely achievable, while < 8% may be appropriate for those with severe hypoglycemia history, limited life expectancy, or when treatment risks outweigh benefits [6]. Four studies in this review used an HbA1c cutoff of 7-7.5% to define glycemic control, while one study used fasting/random blood glucose thresholds [11-15]. ADA guidelines recommend assessing glycemic status using HbA1c or continuous glucose monitoring at least twice annually, with quarterly assessments advised for those not meeting targets, experiencing frequent hypo- or hyperglycemia, or undergoing rapid growth [6]. HbA1c reflects average blood glucose over 2-3 months and is a key marker of long-term control. While these guidelines are standard, their implementation in resource-limited settings such as Ethiopia is challenging due to insufficient equipment, testing materials, and trained personnel. Pediatric diabetes care is not decentralized, and vulnerable populations require targeted attention.

This review identified multiple factors influencing glycemic control. Socio-demographic factors included age, with preschool children generally achieving better control compared to those aged 10-18 years. However, findings were inconsistent, as some studies reported that increasing age was associated with poorer control [11,13]. Lower caregiver education was also linked to poor outcomes; children of caregivers who were illiterate were four times more likely to have poor glycemic control than those whose caregivers had at least secondary education [13]. Behavioral and dietary factors were critical. Consumption of forbidden foods, defined as food items that are not recommended for patients with diabetes except during episodes of hypoglycemia increased the likelihood of poor glycemic control threefold, and children with fewer than three meals per day plus less than two snacks were similarly at higher risk [11]. Treatment-related factors included insulin dosage, adherence, and complications. Each one-unit increase in insulin dosage decreased the odds of poor control by 4%, suggesting dose adjustments can be protective [13]. Treatment discontinuation doubled the risk of poor control, while recent hospital admissions increased risk by 7.9-fold [13]. Lipodystrophy at injection sites and poor glucose monitoring adherence increased risk fourfold and 4.4-fold, respectively [13]. Inability to afford insulin when free supplies were unavailable increased risk sixfold [13]. Caregiver and health system factors were also significant. Children with fathers or guardians as primary caregivers had higher odds of poor control compared to those with mothers (4.4-6 times) [13]. Minimal caregiver supervision during insulin administration increased the odds of poor control by 5.4 times. Problems encountered during hospital visits increased risk by 3.4-fold [13]. In contrast, family involvement in insulin administration and regular blood glucose monitoring were protective.

Comorbid conditions and birth order (being third-born) were also associated with worse control, although some evidence suggested third-born children could benefit from increased parental experience [14]. The duration of diabetes was another important determinant. Children with disease duration >5 years were more likely to have poor control, possibly due to cumulative financial burdens affecting access to insulin and monitoring tools [13,19]. Interestingly, children with diabetes-related complications, such as DKA, retinopathy, or nephropathy, were less likely to have poor glycemic control, potentially due to heightened vigilance following complications [13]. However, this should be interpreted cautiously, as HbA1c reflects only the previous 2-3 months, and chronic complications are typically associated with long-term poor glycemic control [19,20].

### Limitation

This review has several limitations. First, the primary studies included were cross-sectional in design, and the narrative nature of the review precluded pooled effect estimates. Second, there were non-uniform definitions of poor versus good glycemic control across studies, along with an absence of data on type 2 diabetes. Third, the review was restricted to studies published in the English language. Fourth, the effect of anemia and haemoglobinopathies on HbA1c accuracy is not discussed. In addition, a formal risk-of-bias assessment was not performed, however, the included studies were carefully evaluated with respect to study design, sample size, and outcome definitions.

## Conclusion

Poor glycemic control is highly prevalent among Ethiopian children and adolescents with type 1 diabetes mellitus. Several modifiable factors-such as inadequate caregiver education, limited access to insulin, poor treatment adherence, irregular meal patterns, and insufficient blood glucose monitoring-consistently contribute to suboptimal outcomes. Strengthening diabetes education for families, improving access to insulin and home-based monitoring tools, and addressing financial and supply-related barriers are therefore essential. Priority interventions should include expanding the availability of self-monitoring of blood glucose (SMBG), ensuring a reliable and continuous insulin supply, developing adolescent-friendly diabetes services, and enhancing caregiver support systems. In addition, implementing standardized diabetes education programs for families and schools, promoting school-based support and psychosocial counseling, establishing policies for subsidized glucometers and HbA1c testing, and ensuring continuous capacity building for healthcare providers involved in pediatric diabetes care are critical. Furthermore, large-scale, well-designed studies are needed to generate robust evidence to further improve diabetes care and outcomes in children and adolescents in Ethiopia.
